# User-centred development of a monitoring and feedback tool to stimulate physical activity

**Published:** 2012-06-15

**Authors:** Sanne van der Weegen, Renée Verwey, Marieke Spreeuwenberg, Huibert Tange, Trudy van der Weijden, Luc de Witte

**Affiliations:** CAPHRI, Department of Health Services Research Maastricht University, The Netherlands; CAPHRI, Department of Health Services Research Maastricht University, The Netherlands; CAPHRI, Department of Health Services Research Maastricht University, The Netherlands; CAPHRI, Department of General Practice, Maastricht University, The Netherlands; CAPHRI, Department of General Practice, Maastricht University, The Netherlands; CAPHRI, Department of Health Services Research Maastricht University, The Netherlands

**Keywords:** self management, physical activity, persuasive technology, primary care

## Abstract

**Introduction:**

Although physical activity has many positive effects on the quality of life and prognosis, it is very difficult for people with COPD or diabetes type 2 (DM2) to be sufficiently active. Unfortunately, the long-term adherence to several care interventions that try to enhance the physical activity level of these people is generally low. Self-monitoring and feedback integrated in technology might help promoting and sustaining an active lifestyle, especially when it is part of health care services. As a prerequisite, for useful technology and a successful intervention that meets the requirements and preferences of the end users, it is important to involve the end users in the design process at an early stage.

**Aim:**

In this contribution we report how users were involved in the design of an innovative monitoring and feedback tool to support self-management of an active lifestyle for COPD and DM2 patients. This tool will be implemented in primary care in a Self-management Support Program (SSP). Users were involved in particular to elicit:

1. Which feedback they need to optimally support them in their self-management.
2. How this feedback should be presented.
3. How the tool could be designed in order to be attractive, easy to use and suitable to wear on a daily basis.

**Methods:**

An iterative user-centred approach was used to design both the tool and the SSP. Patients, care professionals and technicians were actively involved in the development process. Two patient representatives were added to the research team to help outlining the research strategy. A multidisciplinary team of technicians and computer engineers was established to help deciding on the development of the tool. Sixteen care professionals, 7 COPD and 8 DM2 patients were involved in interviews and focus groups to elicit the functional requirements of the tool.

**Results:**

The conceptual idea of the tool is adapted to the requirements and preferences of the end users. The user-centred design process resulted in a self-management tool that consists of three parts: an accelerometer that monitors physical activity, a smartphone that shows feedback based on this activity and tailored to personal goals, and a server that stores the data and sends the feedback messages to the patients. This server also provides a summary to practice nurses, to prepare them for consultations, telephone calls or email conversations with patients.

**Conclusions:**

The involvement of end users in the development of the tool has led to new insights that increase its acceptability and usability. The tool will soon be validated and tested in pilot studies. The effects of the Self-management Support Program with the tool, embedded in primary care, will be measured in an RCT.

